# Institutes of Surgery: Arranged in the Order of the Lectures Delivered in the University of Edinburgh

**Published:** 1838-02-28

**Authors:** 


### BIBLIOGRAPHICAL NOTICE.

Institutes of Surgery: *Arranged in the order of the Lectures delivered in the University of Edinburgh.* By Sir Charles Bell, K. G. H. F. R. SS. L. & E. Professor of Surgery, &c, &c. &c. *In two volumes; vol.* 1, 12*mo. pp.* 353. *Edinburgh*, 1838.

We expected much from this volume, and we must confess that we have been proportionably disappointed. We anticipated, and we think had almost a right to look for, a complete treatise on the institutes and practice of surgery, embodying the views of the gifted author on all the various subjects connected with his science, and announcing likewise the results of a long and well-tried experience. From the acknowledged abilities and unlimited opportunities of Sir Charles Bell, as well as from the enviable and merited station which he has so long occupied in the view of the surgical world, both as a teacher and practitioner, the profession had strong claims upon him for the production of a work which they might hereafter regard as standard, and refer to with pride and satisfaction, and of such a nature we imagined finding the present undertaking. It is with deep and unaffected regret that candour obliges us to own that the results fall lamentably short of the expectations so reasonably entertained, and that this book is little more than the mere outlines of the lectures delivered at the medical school of Edinburgh. We are the more surprised at this abortion, as in the preface the writer gives us his notions of what he thinks requisite to constitute a good practical surgical treatise, and which are so excellent and just, that few, we suspect, would be disposed to gainsay them.

Throughout the volume before us, the reader will discover many precepts of direct and indisputable utility, marked by justness and sobriety of thought, mingled with much which is of an ambiguous tendency and questionable value. We were prepared for a repetition of old prejudices, and assertions of doubtful utility and worth, clothed in peculiar and characteristic diction, yet many of the doctrines and opinions proclaimed, have startled us from their novelty, vagueness, and inaccuracy, as much as they have offended us by their exclusiveness and dogmatism.

The style is in general forcible, and perhaps rather attractive, but is often abrupt, harsh and inelegant, if not at times positively incorrect.

In his animadversions upon continental surgeons and practice, the author strikes us as one who is more sincere than gentle, and whose frankness and sense of honesty exceed vastly his courtesy and good humour.

What we conceive to be a flagrant and prominent imperfection in this work is, the unpardonable omission of any pathological remarks, reducing it in a great measure to a mere dresser's manual, abounding with rules of routine practice, a certain method of encouraging empiricism, and degrading the science to the level of a mechanical art. The only certain foundation of the therapeutic edifice is a correct knowledge of physiology and pathology—an intimate acquaintance with the functions in a healthy and diseased condition, of their natural as well as their perverted action. Since the morbid anatomy of the internal organs has attracted attention and been prosecuted with becoming ardor and precision, the strides of medicine to more satisfactory curative means have been signal and prodigious, and until external diseases are subjected to the same investigation, the *operator* will, we fear, invariably rank the *surgeon*. Of such vital importance did the late Baron Dupuytren deem surgical pathology, that he bequeathed 300,000 francs for the foundation of a chair of it in the Faculty of Medicine.

This defect strikes us very forcibly in Chapter X, *on Tetanus*, in which the reader will scarcely credit that not one word is said regarding the implication of the nervous system in this formidable affection. Our author thus summarily dismisses that part of his subject: "What produces these terrible symptoms we hardly know. * * * Further we know nothing of the nature of this complaint." p. 84. It may not be out of place to notice here also his comment on the treatment of this disease. "I fear that it is only in the milder forms of the disease that authors have boasted of their success." p. 85. With so crude and imperfect views of the nature of tetanus we are not surprised at the want of individual success which he so candidly acknowledges. In this city, one of our practitioners at least, has been more fortunate in this affection, and his cases were certainly not of very benign aspect. We think that Sir C. Bell might have advantageously consulted the 31st number of the American Journal of the Medical Sciences, p. 24, where he would have found a report of the happy issue of a case of tranmatic tetanus, of by no means of "mild form." The same curative means, we may add, have been since repeated with entire success in three other instances.

Of such expressions as the following, we are at a loss to discover the humour any more than the delicacy. Speaking at p. 316, of an instance of congenital imperfect prepuce he adds, "I cut it up with the point of the bistoury, and the little man ***** and laughed!"

We regret that our limits preclude us from substantiating by any extended analysis, or copious extracts, the opinion that a sense of duty has compelled us to express of this volume, and from an impartial performance of which we have not been deterred by a personal prediliction for the author (which we confess to be strong,) or been restrained by the undue influence of a name. If we have been mistaken in our estimate of Sir Charles Bell's labours, we trust that candour and an earnest desire to uphold and maintain the dignity of surgery, will render venial the fault. We recommend to the profession a perusal of the work, for independent of the omissions and defects, they will find much to amuse as well as to instruct.

Our space will not permit us to do more than cursorily indicate the principal divisions and the headings of the several chapters.

To the first volume is prefixed an introductory chapter on the "Course of Study," which contains much excellent and sensible advice to the surgical student, and exhibits considerable fairness in the appreciations of the abilities of the native contemporaries of the author. There are three grand or principal divisions. 1. The General Principles and External injuries; under this head are included Inflammation, and what are commonly, though inaccurately we conceive, named its terminations; wounds; the various affections and lesions of the bones and joints; and injuries to the head. Division 2. Diseases of the Natural Passages, and the operations performed for their relief. The 22nd chapter, which treats of the diseases of the urethra, is unquestionably more valuable than anything in the work, and is distinguished by clearness of views and soundness of practice. The 3d, and concluding division, treats of the great operations.

